# Clinical Relevance of Unexpected Findings of Post-Mortem Computed Tomography in Hospitalized Patients: An Observational Study

**DOI:** 10.3390/ijerph17207572

**Published:** 2020-10-18

**Authors:** Max G. Mentink, Bartholomeus G. H. Latten, Frans C. H. Bakers, Casper Mihl, Roger J. M. W. Rennenberg, Bela Kubat, Paul A. M. Hofman

**Affiliations:** 1 Department of Radiology and Nuclear Medicine, Maastricht UMC, P. Debyelaan 25, 6229 HX Maastricht, The Netherlands; fch.bakers@mumc.nl (F.C.H.B.); casper.mihl@mumc.nl (C.M.); paul.hofman@mumc.nl (P.A.M.H.); 2 Department of Pathology, Maastricht UMC, P. Debyelaan 25, 6229 HX Maastricht, The Netherlands; bart.latten@mumc.nl (B.G.H.L.); bela.kubat@mumc.nl (B.K.); 3 Netherlands Forensic Institute, Laan van Ypenburg 6, 2497 GB The Hague, The Netherlands; 4 CARIM School for Cardiovascular Diseases, Maastricht University, P. Debyelaan 25, 6229 HX Maastricht, The Netherlands; 5 Department of Internal Medicine, Maastricht UMC, P. Debyelaan 25, 6229 HX Maastricht, The Netherlands; r.rennenberg@mumc.nl

**Keywords:** radiology, post-mortem computed tomography, unexpected findings, autopsy

## Abstract

*Background and objective:* The current literature describing the use of minimally invasive autopsy in clinical care is mainly focused on the cause of death. However, the identification of unexpected findings is equally important for the evaluation and improvement of daily clinical care. The purpose of this study was to analyze unexpected post-mortem computed tomography (PMCT) findings of hospitalized patients and assess their clinical relevance. *Materials and methods:* This observational study included patients admitted to the internal medicine ward. Consent for PMCT and autopsy was requested from the next of kin. Decedents were included when consent for at least PMCT was obtained. Consent for autopsy was not obtained for all decedents. All findings reported by PMCT were coded with an International Classification of Diseases (ICD) code. Unexpected findings were identified and subsequently categorized for their clinical relevance by the Goldman classification. Goldman class I and III were considered clinically relevant. Additionally, correlation with autopsy results and ante-mortem imaging was performed. *Results*: In total, 120 decedents were included and evaluated for unexpected findings on PMCT. Of them, 57 decedents also underwent an autopsy. A total of 1020 findings were identified; 111 correlated with the cause of death (10.9%), 508 were previously reported (49.8%), 99 were interpreted as post-mortem changes (9.7%), and 302 were classified as unexpected findings (29.6%). After correlation with autopsy (in 57 decedents), 24 clinically relevant unexpected findings remained. These findings were reported in 18 of 57 decedents (32%). Interestingly, 25% of all unexpected findings were not reported by autopsy. *Conclusion:* Many unexpected findings are reported by PMCT in hospitalized patients, a substantial portion of which is clinically relevant. Additionally, PMCT is able to identify pathology and injuries not reported by conventional autopsy. A combination of PMCT and autopsy can thus be considered a more comprehensive and complete post-mortem examination.

## 1. Introduction

### 1.1. Background

A search for alternative post-mortem diagnostics has followed the decline in clinical autopsy rates [1]. The literature dedicated to post-mortem imaging and its developments is expanding, especially in the field of forensic medicine [2,3,4,5,6]. However, post-mortem imaging remains underutilized in daily clinical practice, and as a consequence, the literature on the clinical application of post-mortem imaging is limited. Imaging modalities commonly used in post-mortem imaging are computed tomography and magnetic resonance imaging, which can be supplemented by the use of angiography, pulmonary ventilation, or biopsy [3,4,7,8,9]. Among these possibilities, post-mortem computed tomography (PMCT) is considered to be the most feasible imaging modality because it is fast and widely available. So far, three clinical studies have shown that PMCT is in agreement with autopsy on the cause of death of hospitalized patients in 64–74% of all cases [10,11,12]. These studies confirmed that PMCT has a significantly higher agreement on the cause of death than the clinical assessment alone [11,12]. Additionally, it was stated that the detection of cardiovascular diseases (e.g., coronary occlusion or stenosis, pulmonary embolism) is the major limitation of non-enhanced PMCT [7,10,12]. This limitation also explains why post-mortem angiography is a promising technique in the field of post-mortem research [4,13]. Although PMCT has its limitations, there are some advantages to PMCT over autopsy. The literature shows that PMCT visualizes more skeletal pathologies and traumatic injuries than autopsy, as autopsy is limited to certain anatomical cavities and structures, whereas PMCT is not [3,14,15,16].

The current literature on post-mortem imaging is mainly focused on the cause of death, which is an important reason for post-mortem examinations. However, this is not the only relevant parameter. The identification of unexpected findings could also be important to clinicians, because they potentially reveal comorbidities that could have altered the patient’s treatment and potentially affected the prognosis. Such clinically relevant findings are a valuable source of information that enables quality assessment and identification of a potential improvement of clinical care [17,18,19,20]. The literature dedicated to clinically unsuspected or unknown findings of PMCT in hospitalized patients is limited and does not specify the clinical relevance of these findings [6,21].

### 1.2. Purpose

The purpose of the study was to analyze unexpected PMCT findings and their clinical relevance in hospitalized patients.

## 2. Material and Methods

### 2.1. Setting and Design

This single-center, observational study was conducted in a tertiary university hospital. All data were collected from the original PMCT reports stored in the radiology information system (IMPAX RIS 1.3; Agfa, Mortsel, Belgium). Additional patient information (e.g., demographics) was retrieved from the electronic medical records (SAP Netweaver 7.30; SAP SE, Walldorf, Germany). The institutional medical ethics review committee (METC AZM/UM) reviewed the study protocol (reference METC 2017-0260) and subsequently confirmed that official approval by the committee was not required for this study because the Medical Research Involving Human Subjects Act does not apply to this study.

### 2.2. Participants

Patients who died in one of the wards of the Department of Internal Medicine (general internal medicine, gastroenterology, hematology, immunology, geriatrics, nephrology, oncology) during a 23-month period (September 2015 until August 2017) were enrolled prospectively in this study. The treating physicians conducted the consent procedure and an interactive training on the consent procedure was organized prior to the start of the study. The next of kin were asked to give their consent for PMCT, post-mortem biopsy, and autopsy. Additional consent for brain autopsy was also discussed if consent for autopsy was provided. It was possible to give consent for each of the examinations separately as well. Decedents were included when consent for at least PMCT was obtained. Consent for autopsy was not obtained for all included decedents. The available budget determined the sample size.

### 2.3. Procedures

Post-mortem imaging was performed by non-contrast PMCT on the day of death or the next workday. Decedents were scanned with a Somatom Definition Flash (Siemens Healthineers, Forchheim, Germany) or a Brilliance 64 CT scanner (Philips, Best, the Netherlands) with a full-body scan protocol. The images were interpreted and reported by a radiologist with experience in post-mortem radiology using a standardized report template. A resident in pathology, supervised by a pathologist, performed the clinical autopsy according to daily practice. Brain autopsy was only performed if explicit consent was obtained. The radiology reports were made before autopsy results were available. Radiologists and pathologists were not blinded to clinical information or any preceding post-mortem examinations.

### 2.4. Identification and Classification of PMCT Findings

Findings were collected from the PMCT report and classified according to the International Classification of Diseases (ICD, World Health Organization, 10th revision, 2016) [22]. An unexpected PMCT finding was defined as ‘a previously unknown finding or diagnosis based on a pathological process which can be related to, but is not, the cause of death, considering the patient’s clinical history and age’. This definition excluded all expected findings (e.g., cerebral atrophy, degenerative skeletal changes, compression atelectasis in the presence of pleural effusion, air in the urinary bladder in the presence of a catheter), post-mortem changes (gas configurations or redistribution of fluids in the absence of pathology, e.g., intravascular gas, ascites, pleural or pericardial effusion), postoperative changes (e.g., surgical clips, organ removal, prosthesis material), causes of death, and all clinically known diagnoses and previously described findings on ante-mortem imaging. Reports of ante-mortem examinations and the electronic medical records of all departments were comprehensively reviewed by a physician (M.M.) to determine whether a finding had been previously reported. There were no limitations to the accessibility of the decedents’ medical records. Additionally, the findings were correlated with ante-mortem imaging to assess whether a finding was visible on imaging before death and not reported.

Unexpected findings were categorized by their clinical relevance according to the criteria described by Goldman et al. (class I–IV) [23]. A physician (M.M.) assigned the Goldman classes to the findings independently, and in consensus with a pathologist and radiologist for cases that were not straightforward. The cause of death had been determined prior to this study in consensus with the treating physician and a radiologist, and a pathologist when autopsy was performed. Class I represents major diagnoses with a direct relation to the cause of death, the detection of which would have led to changes in management and therapy that could have prolonged survival or cured the patient. Class II represents major diagnoses with a relation to the cause of death, the detection of which would have led to changes in management and therapy, but the adjusted therapy would not have prolonged survival or cured the patient. Class III diagnoses are minor diagnoses with no direct relation to the cause of death, which should have been treated or would have eventually affected the prognosis. Class IV diagnoses are non-diagnosable (occult) diseases with possible genetic or epidemiological significance, but no relation to the cause of death. Classes I and III were defined as clinically relevant because these findings would have affected the patient’s prognosis. The ICD codes of unexpected findings (Goldman class I and III) were used to categorize them into corresponding ICD chapters [22].

An analysis was provided for cases in which autopsy was performed (autopsy group). The findings in these cases were correlated to the results of the autopsy, because autopsy is the current reference standard. The full autopsy reports were available for this correlation. A finding that was reported in the PMCT report as well as the autopsy report was labeled as concordant. Findings that were reported in the PMCT report, but were not described in the autopsy report were interpreted as either false-positive (type I error of PMCT) or false-negative (type II error of autopsy). In order to be interpreted as a false-negative by autopsy, a PMCT finding would have to be either irrefutable (based on imaging) or outside the field of view of autopsy.

### 2.5. Methodological Analysis

For descriptive purposes, nominal and categorical variables were presented as absolute numbers and percentages, and continuous variables as mean (±SD) or median with corresponding IQR. No methodological tests for significance were performed in the analysis. Standard deviations were calculated with SPSS (IBM^®^ SPSS^®^ Statistics for Macintosh, Version 24.0.0.0. Armonk, NY, USA: IBM Corp.).

## 3. Results

### 3.1. Patient Demographics

Of the 123 decedents enrolled in the study, three were excluded because PMCT could not be performed (the corpse had already been transported to the funeral home or the autopsy had already been performed). The PMCTs of 120 decedents were available for analysis: mean age 69 ± 13.9 years, age range 71 (24–95), 73 males, 47 females. Consent for autopsy was provided for 57 of 120 decedents (48%). Brain autopsy was performed in 22 of these decedents (39%). PMCT was performed at a median interval of 16.8 h after death (IQR: 10.9–28.7).

### 3.2. PMCT Findings

In total, 1020 findings were identified in 120 PMCT reports. Of these, 111 findings correlated with the cause of death (10.9%). Some 508 findings had been previously reported (49.8%). Another 99 findings were interpreted as post-mortem changes (99/1020, 9.7%). The most frequent post-mortem changes were pericardial effusion (33/99, 33.3%), fluid-filled paranasal sinus (25/99, 25.3%), ascites (17/99, 17.2%), and aerobilia (10/99, 10.1%). The remaining 302 findings were classified as unexpected findings (29.6%). Table 1 shows how the 1020 PMCT findings are subdivided among the different ICD chapters. In chapter IX, the most frequent findings were coronary sclerosis (89/209, 42.6%), pericardial effusion (49/209, 23.4%), and atherosclerosis (27/209, 12.9%). In chapter X, the most frequent findings were pleural effusion (101/310, 33.6%), lung consolidation (86/310, 27.7%), and sinusitis (40/310, 12.9%). The most frequent findings in chapter XVIII were ascites (55/153, 35.9%), enlarged lymph nodes (49/153, 32%), and anasarca (35/153, 22.9%). Seven skeletal injuries in chapter XIX could be correlated with the autopsy results; five of them had not been described in the autopsy reports.

### 3.3. Relevant Unexpected PMCT Findings

**Figure 1:**
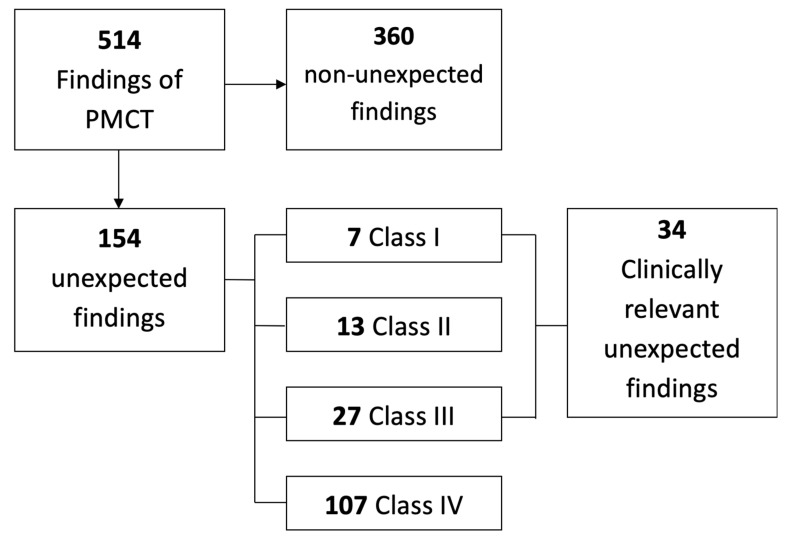
Flowchart of post-mortem computed tomography (PMCT) findings in the autopsy subgroup of 57 decedents. The findings are classified according to Goldman.

In 57 cases, correlation with the autopsy results was possible. A total of 514 findings were reported in this subgroup of decedents, and 154 findings met the criteria of an unexpected finding. Nine of the 154 additional findings (6%) were visible, but not reported on ante-mortem imaging. However, none of these findings were clinically relevant (e.g., enlarged lymph node, pancreatic or prostatic calcification, hyperostosis frontalis interna). The 154 additional findings were classified according to the Goldman classes (Figure 1). Seven and 27 clinically relevant findings were directly (class I) and indirectly (class III) related to the cause of death, respectively.

The 154 unexpected findings can be subdivided into four groups; findings concordant with autopsy (true-positive), false-positive findings of PMCT (type I error), false-negative findings of autopsy (type II error), and findings visualized with PMCT in the absence of brain autopsy (Table 2). Nine of the 38 false-negative unexpected findings of autopsy were outside the field of view of the autopsy (24%). This group of findings contains pathologies of structures that do not form part of the cavities and body region normally assessed during the autopsy (e.g., skeletal injury or lesion, subcutaneous lesion). Because seven clinically relevant unexpected findings were classified as false-positive, and three findings in the brain could not be correlated with the brain autopsy results, a total of 24 clinically relevant unexpected true findings remain. These findings were reported in 18 different decedents, 32% of the autopsy subgroup.

**Table 2:** Clinically relevant unexpected findings divided into four groups of findings: findings concordant with autopsy (true-positive), false-positive findings of PMCT (type I error), false-negative findings of autopsy (type II error), and findings visualized with PMCT in the absence of brain autopsy. For each group, the findings are reported in the last column.

	Unexpected Findings (*n* = 154)	Clinically Relevant Unexpected Findings (*n* = 34)	Reported Clinically Relevant Findings
Concordant with autopsy	92	21	Pancreatitis, pneumonia, excessive pleural fluid, pulmonary edema, gastro-intestinal bleeding, lung bleeding, lung mass, large hematoma (groin), pneumoporta (autopsy showed bowel ischemia), pneumothorax.
False-positive of PMCT (type I error)	21	7	No pathological substrate was found during autopsy in four of these findings (lung consolidation, lung edema, lung bleeding, pancreatitis). The other three findings were a lung consolidation, which turned out to be lung edema; a retroperitoneal bleeding that turned out to be a suppurative pyelonephritis; and pericardial fluid, where autopsy showed a thickened pericardium with adhesions.
False-negative of autopsy (type II error)	38	3	Hydropneumothorax, periprosthetic fracture, pneumatosis intestinalis. Two of these findings are illustrated in Figure 2.
PMCT findings in absence of brain autopsy	3	3	Cerebral mass, multiple cerebral metastases, and one case with post-procedural hypoxia. One of these cases is illustrated in Figure 3.

**Figure 2:**
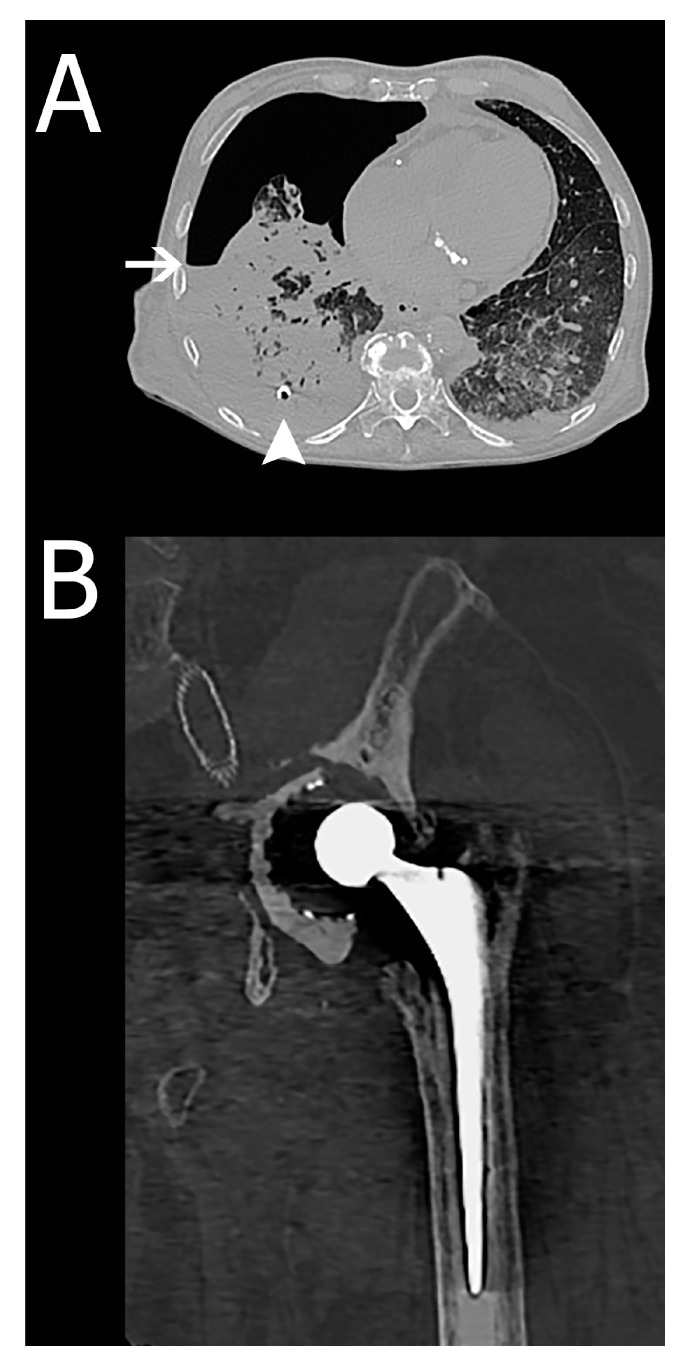
Two cases that show a clinically relevant unexpected finding identified by PMCT and interpreted as false-negative of autopsy. (**A**) This example shows a hydropneumothorax in a 73-year-old woman (class I finding). An air-fluid level can be identified at the white arrow. The pleural drain that was placed for drainage of pleural fluid can also be seen (white arrowhead). The pneumothorax component was unknown, and the pneumothorax test during autopsy was negative. (**B**) A 74-year-old male with a periprosthetic fracture of a hip prosthesis. The autopsy report mentioned a normal position and mobility of the extremities with no fractures. The autopsy determined the cause of death as a pneumosepsis. The finding did not have a direct relationship to the cause of death and was subsequently classified as a class III finding.

**Figure 3:**
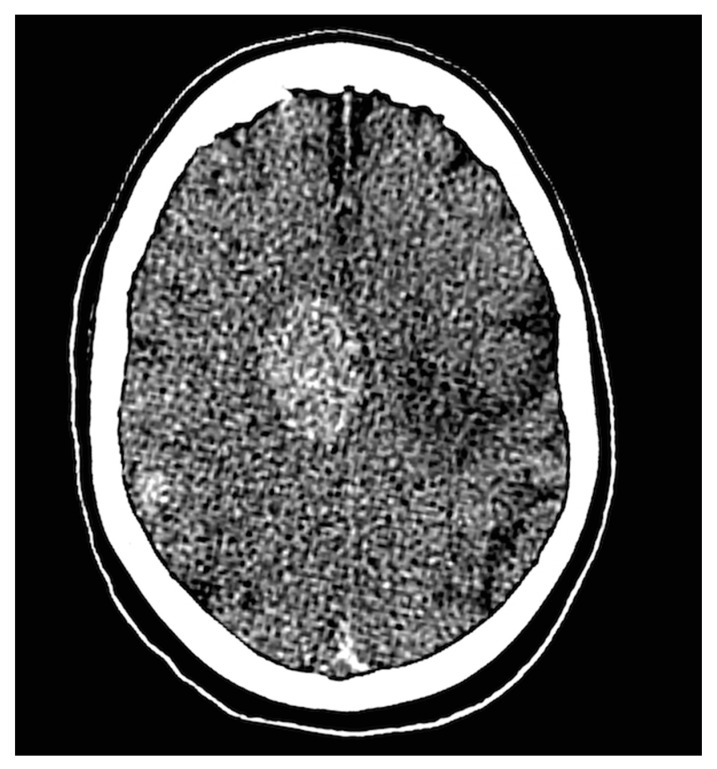
This figure shows multiple dense intra-cranial masses in the right hemisphere identified by PMCT in a 67-year-old male recently diagnosed with a stage IV small-cell lung carcinoma. The patient experienced no neurological complaints and showed no abnormalities during the neurological examination. The intra-cranial masses were suspected to be cerebral metastases and subsequently scored as a class III unexpected finding. Brain autopsy was not performed.

## 4. Discussion

Our study presents an overview of findings that can be expected with a non-enhanced PMCT in hospitalized patients. Of all reported PMCT findings, 10% were post-mortem changes, 11% correlated with the causes of death, 50% were known findings, and the remaining 29% were classified as unexpected findings. Clinically relevant unexpected findings were reported in 32% of all decedents. Multiple unexpected findings (38/154, 25%) were not reported by autopsy, three of which were clinically relevant findings. Our findings raise the question of whether a post-mortem examination consisting of autopsy only is truly the rightful reference standard.

The literature on unexpected PMCT findings unrelated to the cause of death is limited. Wichmann et al. described 10 new major (Goldman class I or II) and 53 new minor diagnoses (Goldman class III or IV) identified by multidetector CT in a cohort of 47 hospitalized patients at the intensive care unit [21]. Similar to our study, most findings were made in the cardiovascular and respiratory system. Moreover, the incidence of new findings reported by Wichmann et al. (63/236, 27%) is very similar to our results (154/514, 30%). The clinical relevance of these findings was not assessed. Wichmann et al. reported 13 missed fractures on autopsy, which is more than those observed in our patient population. The most likely explanation for this discrepancy is that patients from the intensive care units have a higher incidence of skeletal injuries (after trauma or resuscitation) than patients included from the Department of Internal Medicine. This finding is illustrative of the additional value of PMCT, however, because skeletal injuries can be clinically relevant. Although the results are comparable, the clinical relevance of reported (new) findings was not determined, which is an essential step in the interpretation of the added value of PMCT. Clinically relevant missed or unknown findings are the core of quality improvement. Such findings need to be identified before one can learn from them. Our study is the first to report on the relevance of such unexpected findings and the rate at which they can be detected by PMCT. These findings were discussed during the multidisciplinary mortality review board. The clinical relevant unexpected findings led to both educational and quality improvement discussions.

The main limitation of this study is the consent rate for autopsy, which is a known difficulty in the field of post-mortem research. Autopsy was performed in less than half of the cases (57 of 120 decedents). This finding indicates that consent for a non-invasive post-mortem examination is obtained from the next of kin more easily than consent for autopsy. Similarly, consent for brain autopsy was provided in only 22 of 57 autopsies. These consent rates limited the number of cases in which PMCT findings could be correlated with the current reference standard. In contrast to autopsy, PMCT has no restrictions concerning the skull, which is a major benefit of PMCT over autopsy. The added value of PMCT, by means of its unlimited coverage of anatomical regions, is illustrated by two cases for which PMCT reported one or multiple previously unknown intracranial masses, but no consent for brain autopsy was provided. These findings would have remained unknown if PMCT had not been performed. One of these cases is illustrated in Figure 3. Another limitation is that radiologists and pathologists were not blinded for clinical information because this study was conducted in a clinical setting. However, regarding the unexpected findings, this had no influence on the results and methodological quality because the unexpected findings identified by PMCT were, by definition, unknown to the radiologist and pathologists at the time of the examinations. A characteristic that contributes to the strength of this study is its external validity, which is high because this study reflects the results representative of daily clinical practice. Strict definitions of unexpected and clinically relevant findings were maintained to allow this research to be reproduced by other groups.

The use of PMCT is not standard in clinical medicine. Several advantages are clear, such as its non-invasive character, relatively high consent rate, unlimited coverage of anatomical regions, and images that can be re-interpreted. However, its implications for daily use in addition to autopsy are not yet fully known. Multiple studies have reported on the agreement with autopsy on the cause of death, but no studies have been published on the clinical relevance of unexpected findings reported by PMCT [10,11,12]. PMCT and autopsy are fundamentally different examinations, thus PMCT has a different way of visualizing pathological processes. Abnormal air configurations, skeletal pathology or injury, calcifications, and fluid collections are more easily identified on cross-sectional imaging than during autopsy, as is also illustrated by several cases shown in Figure 2, in accordance with the published literature [3,14,15,16]. However, non-enhanced PMCT has limitations as well, for instance, visualization of cardiovascular pathologies (i.e., coronary occlusion or stenosis and pulmonary embolism) that cannot be visualized without intravascular contrast. As both techniques have their strengths and weaknesses that complement each other, a combination of the two could be considered a more comprehensive post-mortem examination. With this study, we hope to add to the growing evidence on PMCT and post-mortem imaging in general. The future might entail a more frequent use of post-mortem imaging in order to gain new insights and enable quality control and improvement in a non-invasive manner. Ethical considerations and local legislation should be extensively deliberated before implementing such techniques and consent by family members should always be sought.

## 5. Conclusions

Post-mortem CT has several unique advantages over conventional autopsy, such as its non-invasive character. The cause of death is not the only finding during a post-mortem examination. Many unexpected findings are reported, a substantial portion of which are clinically relevant. Clinically relevant unexpected findings were reported in 32% of all decedents. Additionally, PMCT is able to identify pathology and injuries not reported by conventional autopsy. A combination of these two post-mortem examinations can thus be considered more complete.

## Figures and Tables

**Table 1:** An overview of all post-mortem computed tomography (PMCT) findings categorized in their corresponding International Classification of Diseases (ICD) chapter. Chapters V, VII, XV, XVI, XX, and XXI are not shown because no findings were reported for these chapters. The number of findings and the percentage of the total number of findings are presented. Additionally, the number of unexpected findings in the chapters is shown, and as a percentage of total findings in the corresponding chapter. For example, 22 PMCT findings that correlate with a disease in the nervous system were reported, which is 2.2% of the total number of PMCT findings in 120 decedents. Four of those 22 findings were unexpected, which is 18.2% of all findings of the nervous system.

ICD Chapter	Definition of the ICD Chapter	Number of Findings *n* (%)	Number of Unexpected Findings in the Chapter *n* (%)
I	Infectious and parasitic diseases	6 (0.6)	4 (66.7)
II	Neoplasms	53 (5.2)	15 (28.3)
III	Diseases of the blood and blood-forming organs and certain disorders involving the immune mechanism	1 (0.1)	0 (0)
IV	Endocrine, nutritional, and metabolic diseases	21 (2.1)	16 (76.2)
VI	Diseases of the nervous system	22 (2.2)	4 (18.2)
VIII	Diseases of the ear and mastoid process	2 (0.2)	1 (50)
IX	Diseases of the circulatory system	209 (20.5)	42 (20.1)
X	Diseases of the respiratory system	310 (30.4)	76 (24.5)
XI	Diseases of the digestive system	107 (10.5)	36 (33.6)
XII	Diseases of the skin and subcutaneous tissue	2 (0.2)	0 (0)
XIII	Diseases of the musculoskeletal system and connective tissue	33 (3.2)	13 (39.4)
XIV	Diseases of the genitourinary system	69 (6.8)	22 (31.9)
XVII	Congenital malformations, deformations, and chromosomal abnormalities	10 (1.0)	3 (30)
XVIII	Symptoms, signs, and abnormal findings, not elsewhere classified	153 (15.0)	61 (39.9)
XIX	Injury, poisoning, and certain other consequences of external causes	22 (2.2)	9 (40.9)
Total		1020 (100)	302 (29.6)
